# Genome Sequence of the Cheese-Starter Strain *Lactobacillus delbrueckii* subsp. *lactis* CRL 581

**DOI:** 10.1128/genomeA.00602-13

**Published:** 2013-08-08

**Authors:** Elvira María Hebert, Raúl R. Raya, Lucía Brown, Graciela Font de Valdez, Graciela Savoy de Giori, María Pía Taranto

**Affiliations:** Centro de Referencia para Lactobacilos (CERELA-CONICET), San Miguel de Tucumán, Argentina

## Abstract

We report the genome sequence of *Lactobacillus delbrueckii* subsp. *lactis* CRL 581 (1,911,137 bp, GC 49.7%), a proteolytic strain isolated from a homemade Argentinian hard cheese which has a key role in bacterial nutrition and releases bioactive health-beneficial peptides from milk proteins.

## GENOME ANNOUNCEMENT

*Lactobacillus delbrueckii* subsp. *lactis* and *L. delbrueckii* subsp. *bulgaricus* are homofermentative thermophilic lactic acid bacteria (LAB) widely used as starter cultures for the manufacture of a variety of fermented dairy products, such as fermented sour milks and Swiss and Italian type cheeses (1). To date, the genome analyses of *L. delbrueckii* subsp. *bulgaricus* strains ATCC 11842, 2038, NDO2, and ATCC BAA-365 have been published (2–6). However, the genome analysis of *L. delbrueckii* subsp. *lactis* has not been published.

*L. delbrueckii* subsp. *lactis* strain CRL 581, isolated from a homemade Argentinian hard cheese (1), possesses an efficient proteolytic system which has a key role in bacterial nutrition as well as contributing to the development of the organoleptic properties of fermented milk products and releasing bioactive health-beneficial peptides (i.e., anti-inflammatory, antihypertensive, and phosphopeptides) from milk proteins (7, 8). The proteolytic system of the CRL 581 strain provides opportunities for the development of novel functional food with potential health-promoting properties. Here, we describe the genome sequence of *L. delbrueckii* subsp. *lactis* CRL 581.

Whole-genome sequencing of *L. delbrueckii* subsp. *lactis* CRL 581 was performed with a 454 GS Titanium pyrosequencer at INDEAR, Argentina. Genomic libraries containing 8-kb inserts were prepared, and 122,806 paired-end reads and 221,348 single-end reads were generated using the 454 GS system, giving 42-fold coverage of the genome. Approximately 99.2% of these reads were assembled into 16 large scaffolds, including 163 nonredundant contigs, using version 2.6 of the 454 Newbler assembler (454 Life Sciences, Branford, CT). The draft genome is a single circular chromosome of 1,911,137 bases in length, with a mean GC content of 49.7%; no plasmids were observed in this genome. Genome annotation was performed by use of the standard operating procedures (SOPs) for prokaryotic annotation from ISGA (9), the RAST annotation server (10), the Glimmer 3.02 modeling software package (11), tRNAscan-SE 1.21 (12), and RNAmmer 1.2 (13). A total of 1,880 coding sequences (CDS), 37 structural tRNAs, and 3 rRNA operons were predicted. There are 254 RAST subsystems represented in the chromosome. Additionally, no functional prophages were identified, although several genes for transposases and two clusters of regularly interspaced short palindromic repeats (CRISPRs) and two potential CRISPRs were found.

The genome of *L. delbrueckii* subsp. *lactis* CRL 581 encodes several components of the proteolytic system, including the cell envelope-associated proteinase (PrtL), aminopeptidases (PepC, PepN, PepM, and PepA), endopeptidases (PepO and PepF), dipeptidases (PepD and PepV), tripeptidase (PepT), and proline peptidases (PepX, PepI, PepP and PepQ) as well as the di/tripeptide Dpp and the oligopeptide Opp systems. The presence of these enzymes supports the role of the cheese starter culture *L. delbrueckii* subsp. *lactis* CRL 581 in milk.

Comparative genome analysis of *L. delbrueckii* subsp. *lactis* CRL 581 with the closely related *L. delbrueckii* subsp. *bulgaricus* strains revealed that they are highly similar. However, proteins and enzymes involved in galactose, sucrose, maltose, and trehalose utilization are present only in the *L. delbrueckii* subsp. *lactis* CRL 581 genome.

This is the first report of the genome sequence of a technologically relevant *L. delbrueckii* subsp. *lactis* strain; these data will be useful for exploration of its biotechnological properties.

### Nucleotide sequence accession numbers.

The data from this whole-genome shotgun project were deposited in GenBank under the accession number ATBQ00000000. The version described in this paper is version ATBQ00000000.1.
